# Editorial: Evolving Robotic Morphologies

**DOI:** 10.3389/frobt.2022.874853

**Published:** 2022-04-14

**Authors:** David Howard, Kyrre Glette, Nick Cheney

**Affiliations:** ^1^ Commonwealth Scientific and Industrial Research Organisation (CSIRO), Brisbane, QLD, Australia; ^2^ RITMO, Department of Informatics, University of Oslo, Oslo, Norway; ^3^ University of Vermont, Burlington, VT, United States

**Keywords:** evolutionary robotics, co-evolution, evolutionary algoritchm, morphology, embodied cognition

Morphological adaptation is a key challenge for Evolutionary Robotics (Bongard, 2013; Doncieux et al., 2015), and robotics in general. Consideration of a robot’s physical form in addition to its control system holds particular promise for imbuing robotic systems with task and environmental adaptability (Sims, 1994; Lipson and Pollack, 2000). The additional degrees of behavioural freedom that can be unlocked when morphology is considered in the evolutionary process allows access to a wide design space, providing a pathway towards resilient, rugged, and adaptable robots that can cope with unforeseen circumstances and be realistically deployed in real-world scenarios (Nygaard et al., 2021b), with corresponding real-world impacts.

Morphology evolution allows us to create and study systems based on the principles of embodied cognition, whereby coupling (evolved) controllers to (evolved) physical substrates in a meaningful environment (Miras and Eiben, 2019) allows for surprising, rich behaviours that emerge from interactions between the three (Lehman and Stanley, 2011; Lehman et al., 2020). Embodied cognition provides a route towards improving the complexity and adaptivity of evolved behaviours, leading to a corresponding increase in the applicability of evolutionary robotics systems to challenging scenarios.

Systems that can fully exploit the principles of embodied cognition and automatically evolve environment-adapted and task-specific robots is an ambitious and challenging endeavour. To date, evolutionary robotics research is largely conducted on morphologically static robots, mainly due to difficulties in either developing encodings that can effectively navigate the expanded, coupled search space of morphology-plus-controller (Faíña et al., 2013; Cheney et al., 2016), and fabricating/assessing morphologically adaptable robotic hardware.

A recent trend has seen implementations of morphological evolutionary robotics for challenging design domains, which has been shown to be effective in designing robots that are, for example, multi-material, soft (Cheney et al., 2014; Fitzgerald et al., 2021), or physically-embodied (Nygaard et al., 2021a). Emerging technologies offer the opportunity to push the boundaries of possibility for morphology evolution, and numerous recent examples from the literature harness principles including multi-material 3D printing (Howard et al., 2021) and advanced modular systems, alongside frameworks (Eiben and Smith, 2015; Howard et al., 2019) that allow researchers to operate in this rapidly-developing scientific field.

This Research Topic called for contributions that illustrate and discuss innovative methods, technologies, software, and philosophical viewpoints that advance the field of evolving robotic morphologies. In the end, the topic accepted 7 original research articles and one mini-review, for a total of 8 accepted articles involving seventeen authors.

In Nordmoen et al. , Nordmoen, Veenstra, Ellefsen and Glette compare three different evolutionary algorithms on the challenging task of optimizing morphology-controller pairings for modular robotics. They show that the quality-diversity algorithm MAP-Elites (Mouret and Clune, 2015) finds the highest-performing solutions and generates the largest morphological diversity, the latter being an advantage when transferring the population to new and more difficult environments. Further, a genealogical ancestry analysis shows that MAP-Elites produces more diverse and higher performing stepping stones than the objective-based search algorithms.

Miras presents Miras, which moves beyond performance-based influences of encodings in evolutionary robotics to consider how genetic encodings constrain the space of robot phenotypes and robot behavior. In particular, the author highlights how two different generative encodings lead to very different attainable robotic design spaces, thus raising awareness about robot encoding biases. The trade-offs between different biases are also scrutinized in light of morphological, control, and behavioral traits.

Talamini et al. , presented by Talamini, Medvet, and Nichele, propose a task-agnostic approach for automatically designing adaptable soft voxel-based robotic morphologies in simulation, based on the concept of criticality. Criticality is a property belonging to dynamical systems close to a phase transition between the ordered and the chaotic regime. They introduce a measure of criticality in the context of voxel-based soft robots based on the concept of avalanche analysis, often used to assess criticality in biological and artificial neural networks. They allow the robots to evolve towards criticality, and confirm that criticality is a useful indicator for adaptability in this context.

Hawthorne-Madell, Aaron, Livingston and Long present Hawthorne-Madell er al. , which explores mechanisms that create genetic variation without the disruption of adapted genes and genomes caused by random mutation. They focus on epigenetic error, a developmental process that acts on materials and parts expressed by the genome. In their system of embodied computational evolution, simulated within a physics engine, epigenetic error was instantiated in an explicit genotype-to-phenotype map as transcription error at the initiation of gene expression. They conclude that random developmental processes offer an additional mechanism for exploration by increasing genetic variation in the face of steady, directional selection. The same authors investigate the role of various intertwined evolutionary processes in the evolution of complex morphologies in Aaron et al. Techniques from evolutionary biology, selection gradient analysis, and morphospace walks are used to assess robot morphologies, with a focus on three evolutionary mechanisms (randomness, development, and selection) in the context of evolving biorobots. The authors demonstrate that the analytical approaches provide valuable insight into evolutionary processes.

In Chand and Howard, Chand and Howard present the first instantiation of the Multi-Level Evolution (MLE) paradigm for automatically defined modular robot evolution. MLE is a decomposition-based combinatorial approach for the generation of reusable libraries of robot components which are combined into high-performing robots. Their proof of concept system combines levels of material, component, and robot discovery, implemented in MAP-Elites. Quality-diversity algorithms at each level allow for the discovery of a wide variety of reusable elements. The results strongly support the initial claims for the benefits of MLE, allowing for the discovery of designs that would otherwise be difficult to achieve with conventional design paradigms.

Moreno and Faiña, by Moreno and Faiña, presents a platform for evolution of morphology in full cycle reconfigurable hardware. The EMERGE (Easy Modular Embodied Robot Generator) implements all three parts necessary to realise a full cycle evolutionary robotics process, i.e., assembling the modules in morphologies, testing the morphologies, and disassembling modules. The mechanical design of the EMERGE module is presented, together with extensive tests. To test the performance of real EMERGE modules, 30 different morphologies are evolved in simulations and subsequently transferred to the real world. Module tracking combined with easy assembly and disassembly enable EMERGE modules to be also reconfigured using an external robotic manipulator. Experiments demonstrate the assembly and disassembly of modules from a morphology.

Finally, the research topic is tied together by Eiben, who presents a critical mini-review entitled Eiben, discussing the challenges faced in the real-world instantiation of phyiscal evolving robotic systems. Eiben identifies multiple enablers which, combined, bring the vision of real-world morphology-controller evolution closer to reality. He notes that sample-efficient evolution is one of the key prerequisites for such systems. Finally, the importance of not only building but also understanding evolving robot systems is emphasised, stating that in order to have the technology work we also need the science behind it.

Overall, the topic consolidates the current state of the art in the field, as well as describing potential future paths of fruitful research. Commonly-noted features include soft robotics, quality-diversity, real-world evolution, and bioinspiration. We hope that this Special Topic is both insightful and inspirational to the field as we continue to bring real-world evolutionary robotics systems to reality.
